# Methicillin-Resistant *Staphylococcus aureus* (MRSA) Detected at Four U.S. Wastewater Treatment Plants

**DOI:** 10.1289/ehp.1205436

**Published:** 2012-09-06

**Authors:** Rachel E. Rosenberg Goldstein, Shirley A. Micallef, Shawn G. Gibbs, Johnnie A. Davis, Xin He, Ashish George, Lara M. Kleinfelter, Nicole A. Schreiber, Sampa Mukherjee, Amir Sapkota, Sam W. Joseph, Amy R. Sapkota

**Affiliations:** 1 Maryland Institute for Applied Environmental Health, University of Maryland School of Public Health, College Park, Maryland, USA; 2 Department of Plant Science and Landscape Architecture and Center for Food Safety and Security Systems, University of Maryland, College Park, Maryland, USA; 3 Department of Environmental, Agricultural & Occupational Health, College of Public Health, University of Nebraska Medical Center, Omaha, Nebraska, USA; 4 Center for Veterinary Medicine, U.S. Food and Drug Administration, Laurel, Maryland, USA; 5 Department of Epidemiology and Biostatistics, University of Maryland School of Public Health, College Park, Maryland, USA; 6 Department of Cell Biology and Molecular Genetics, University of Maryland College Park, College Park, Maryland, USA

**Keywords:** antibiotic resistance, community-acquired methicillin-resistant *Staphylococcus aureus*, methicillin-resistant *Staphylococcus aureus*, methicillin-susceptible *Staphylococcus aureus*, MRSA, MSSA, reclaimed wastewater, wastewater, wastewater treatment plant

## Abstract

Background: The incidence of community-acquired methicillin-resistant *Staphylococcus aureus* (CA-MRSA) infections is increasing in the United States, and it is possible that municipal wastewater could be a reservoir of this microorganism. To date, no U.S. studies have evaluated the occurrence of MRSA in wastewater.

Objective: We examined the occurrence of MRSA and methicillin-susceptible *S. aureus* (MSSA) at U.S. wastewater treatment plants.

Methods: We collected wastewater samples from two Mid-Atlantic and two Midwest wastewater treatment plants between October 2009 and October 2010. Samples were analyzed for MRSA and MSSA using membrane filtration. Isolates were confirmed using biochemical tests and PCR (polymerase chain reaction). Antimicrobial susceptibility testing was performed by Sensititre® microbroth dilution. Staphylococcal cassette chromosome *mec* (SCC*mec)* typing, Panton-Valentine leucocidin (PVL) screening, and pulsed field gel electrophoresis (PFGE) were performed to further characterize the strains. Data were analyzed by two-sample proportion tests and analysis of variance.

Results: We detected MRSA (*n* = 240) and MSSA (*n* = 119) in 22 of 44 (50%) and 24 of 44 (55%) wastewater samples, respectively. The odds of samples being MRSA-positive decreased as treatment progressed: 10 of 12 (83%) influent samples were MRSA-positive, while only one of 12 (8%) effluent samples was MRSA-positive. Ninety-three percent and 29% of unique MRSA and MSSA isolates, respectively, were multidrug resistant. SCC*mec* types II and IV, the *pvl* gene, and USA types 100, 300, and 700 (PFGE strain types commonly found in the United States) were identified among the MRSA isolates.

Conclusions: Our findings raise potential public health concerns for wastewater treatment plant workers and individuals exposed to reclaimed wastewater. Because of increasing use of reclaimed wastewater, further study is needed to evaluate the risk of exposure to antibiotic-resistant bacteria in treated wastewater.

*Staphylococcus aureus* is a bacterial pathogen associated with a wide range of human infections, including skin infections, pneumonia, and septicemia (Bassetti et al. 2009). Infections with this microorganism can be difficult to treat because the strains are often resistant to one or more antibiotics, including methicillin. Methicillin-resistant *S. aureus* (MRSA) was first isolated in 1960, and for the past four decades MRSA infections have been largely associated with hospital environments and referred to as hospital-acquired MRSA (HA-MRSA) (Bassetti et al. 2009; Gorwitz et al. 2008). However, in the late 1990s, community-acquired MRSA (CA-MRSA) infections began to appear in otherwise healthy people who had no known risk factors for these infections (Bassetti et al. 2009; Gorak et al. 1999). The incidence of CA-MRSA has continued to increase in the United States. Outbreaks of CA-MRSA have occurred among individuals sharing close contact with others in schools, prisons, and locker rooms, but other possible environmental reservoirs of MRSA have yet to be comprehensively explored (Diekema et al. 2001).

Identifying environmental reservoirs of MRSA in the community is critical if the spread of CA-MRSA infections is to be controlled. Of other potential environmental reservoirs, wastewater has been identified as a possible source of exposure to MRSA in the community (Börjesson et al. 2009, 2010; Plano et al. 2011). Colonized humans shed MRSA from the nose, feces, and skin; therefore, MRSA can end up in municipal wastewater streams (Börjesson et al. 2009, 2010; Plano et al. 2011; Wada et al. 2010). Börjesson et al. (2009) recently detected MRSA resistance genes in all treatment steps at a Swedish municipal wastewater treatment plant (WWTP). These authors also cultured MRSA from influent samples (Börjesson et al. (2009), as well as influent and activated sludge samples (Börjesson et al. 2010). Currently, as water shortages expand, treated municipal wastewater is increasingly used for applications including landscape and crop irrigation, groundwater recharge, and snowmaking (Levine and Asano 2004; Tonkovic and Jeffcoat 2002). During these activities, individuals applying, using, or coming in contact with reclaimed wastewater could potentially be exposed to MRSA and other bacteria that may remain in treated wastewater (Iwane et al. 2001).

To our knowledge, no studies have demonstrated the occurrence of MRSA in wastewater in the United States. In the present study, we evaluated the occurrence of MRSA and methicillin-susceptible *S. aureus* (MSSA) at four WWTPs located in two different regions of the United States: the Mid-Atlantic region and the Midwest. To further assess the MRSA strains, isolates were characterized by staphylococcal cassette chromosome *mec* (SCC*mec*) typing and pulsed field gel electrophoresis (PFGE), and screened for Panton-Valentine leucocidin (PVL), an exotoxin often associated with virulent strains of *S. aureus.*

## Materials and Methods

**Figure 1:**
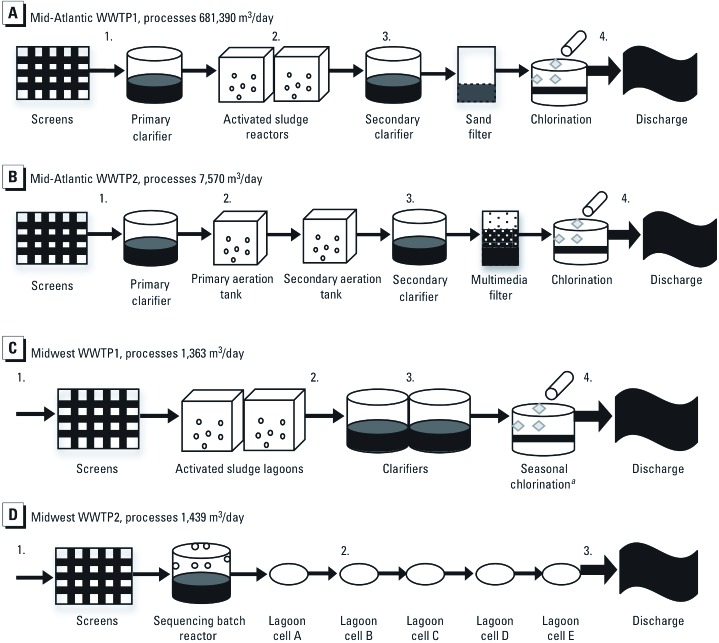
Schematic of wastewater treatment processes at four wastewater treatment plants in the Mid-Atlantic and Midwest regions of the United States. For Mid-Atlantic WWTP1 (*A*) and Mid-Atlantic WWTP2 (*B*), 1 = influent, 2 = activated sludge reactor, 3 = post aeration, and 4 = effluent. (*C*) For Midwest WWTP1, 1 = influent, 2 = post aeration, 3 = secondary clarifier, and 4 = effluent. (*D*) For Midwest WWTP2, 1 = influent, 2 = cell B, and 3 = effluent. ***^a^***Seasonal chlorination takes place in June, July, and August.

*Study sites.* Four WWTPs were included in this study: two in the Mid-Atlantic region and two in the Midwest. The treatment steps and sampling locations at each of the treatment plants are illustrated in Figure 1.

Mid-Atlantic WWTP1 (Figure 1A) is a tertiary WWTP in an urban area that processes 681,390 m^3^/day of wastewater, with a peak capacity of 1.51 million m^3^/day. Mid-Atlantic WWTP2 (Figure 1B), a tertiary WWTP in a suburban area, processes 7,570 m^3^/day of wastewater and has a peak capacity of 45,425 m^3^/day. Tertiary wastewater treatment includes primary treatment (physical removal of solids), secondary treatment (biological treatment), and additional treatment that can include, but is not limited to, chlorination, ultraviolet radiation, or filtration. The incoming wastewater (influent) at both Mid-Atlantic plants includes domestic and hospital wastewater, and effluent (discharge) from both Mid-Atlantic plants is piped to landscaping sites for reuse in spray irrigation.

Midwest WWTP1 (Figure 1C) is a tertiary WWTP in a rural area that processes 1,363 m^3^/day of wastewater, with a peak capacity of 10,978 m^3^/day. The incoming water includes domestic wastewater and agriculturally influenced stormwater. Seasonal chlorination occurs in June, July, and August, and chlorinated effluent is piped to a landscaping site for reuse in spray irrigation. Midwest WWTP2 (Figure 1D), a secondary WWTP (with no on-site disinfection) in a rural area, processes 1,439 m^3^/day and has a peak capacity of 7,571 m^3^/day. Secondary wastewater treatment includes only primary treatment (physical removal of solids) and secondary treatment (biological treatment). The incoming water at this plant includes domestic wastewater, wastewater from a food production facility, and agriculturally influenced stormwater. Unchlorinated effluent is piped to an agricultural site for crop irrigation.

*Sample collection.* A total of 44 grab samples were collected between October 2009 and October 2010: 12 samples from Mid-Atlantic WWTP1; 8 from Mid-Atlantic WWTP2; 12 from Midwest WWTP1; and 12 from Midwest WWTP2. The timing of each sampling event was determined by the availability and schedule of the WWTP operators. The sampling time schedule and specific sampling locations for each plant are indicated in Tables 1 and 2 and Figure 1. Samples were collected in 1-L sterile polyethylene Nalgene® Wide Mouth Environmental Sample Bottles (Nalgene, Lima, OH), labeled, and transported to the laboratory at 4°C. All samples were processed within 24 hr.

**Table 1:** Distribution of MRSA-positive and ‑negative wastewater samples at all WWTPs by sampling event and sampling location.

Sampling location (total samples collected)	Mid-Atlantic WWTP1 (*n* = 12)			Mid-Atlantic WWTP2 (*n* = 8)		Midwest WWTP1 (*n* = 12)			Midwest WWTP2 (*n* = 12)				Total positive samples (%)
	Oct 2009	Dec 2009A	Dec 2009B	Oct 2010A	Oct 2010B	Jul 2010	Sep 2010	Oct 2010	Jul 2010	Aug 2010	Sep 2010	Oct 2010	
Influent (n = 12)	Pos	Pos	Pos	Pos	Pos	Neg	Pos	Pos	Pos	Pos	Neg	Pos	10/12 (83)
Activated sludge reactor (n = 5)	Pos	Pos	Pos	Pos	Pos	—	—	—	—	—	—	—	5/5 (100)
Post aeration (n = 3)	—	—	—	—	—	Neg	Pos	Pos	—	—	—	—	2/3 (67)
Cell B (n = 4)	—	—	—	—	—	—	—	—	Neg	Neg	Neg	Neg	0/4 (0)
Secondary clarifier (n = 8)	Neg	Pos	Pos	Neg	Neg	Pos	Neg	Pos	—	—	—	—	4/8 (50)
Effluent (n = 12)	Neg	Neg	Neg	Neg	Neg	Neg	Neg	Posa	Neg	Neg	Neg	Neg	1/12 (8)
Total positive samples (%)	2/4 (50)	3/4 (75)	3/4 (75)	2/4 (50)	2/4 (50)	1/4 (25)	2/4 (50)	4/4 (100)	1/3 (33)	1/3 (33)	0/3 (0)	1/3 (33)	22/44 (50)
Abbreviations: Neg, negative sample; Pos, positive sample. Samples were collected twice during December 2009 at Mid‑Atlantic WWTP1 (A and B) and twice during October 2010 at Mid-Atlantic WWTP2 (A and B). aSample was collected when chlorination of effluent was not taking place.																										

**Table 2:** Distribution of MSSA-positive and ‑negative wastewater samples at all WWTPs by sampling event and sampling location.

Sampling location (total samples collected)	Mid-Atlantic WWTP1 (*n* = 12)			Mid-Atlantic WWTP2 (*n* = 8)		Midwest WWTP1 (*n* = 12)			Midwest WWTP2 (*n* = 12)				Total positive samples (%)
	Oct 2009	Dec 2009A	Dec 2009B	Oct 2010A	Oct 2010B	Jul 2010	Sep 2010	Oct 2010	Jul 2010	Aug 2010	Sep 2010	Oct 2010	
Influent (n = 12)	Pos	Pos	Pos	Pos	Pos	Pos	Neg	Pos	Pos	Pos	Neg	Pos	10/12 (83)
Activated sludge reactor (n = 5)	Pos	Pos	Pos	Pos	Pos	—	—	—	—	—	—	—	5/5 (100)
Post aeration (n = 3)	—	—	—	—	—	Pos	Pos	Pos	—	—	—	—	3/3 (100)
Cell B (n = 4)	—	—	—	—	—	—	—	—	Pos	Neg	Neg	Neg	1/4 (25)
Secondary clarifier (n = 8)	Neg	Pos	Pos	Neg	Neg	Pos	Neg	Pos	—	—	—	—	4/8 (50)
Effluent (n = 12)	Neg	Neg	Neg	Neg	Neg	Neg	Posa	Posa	Neg	Neg	Neg	Neg	2/12 (17)
Total positive samples (%)	2/4 (50)	3/4 (75)	3/4 (75)	2/4 (50)	2/4 (50)	3/4 (75)	2/4 (50)	4/4 (100)	2/3 (67)	1/3 (33)	0/3 (0)	1/3 (33)	24/44 (55)
Abbreviations: Neg, negative sample; Pos, positive sample. Samples were collected twice during December 2009 at Mid‑Atlantic WWTP1 (A and B) and twice during October 2010 at Mid-Atlantic WWTP2 (A and B). aSamples were collected when seasonal chlorination was not taking place.																										

*Isolation.* Membrane filtration was used to recover *S. aureus* and MRSA from wastewater samples. Briefly, 300 mL of each sample were vacuum filtered through a 0.45-µm, 47-mm mixed cellulose ester filter (Millipore, Billerica, MA). Filters were then enriched in 40 mL of m Staphylococcus broth (Becton, Dickinson and Company, Franklin Lakes, NJ), vortexed, and incubated at 37°C for 24 hr. A 10-µL loopful of each enrichment was then plated in duplicate on MRSASelect (Bio-Rad Laboratories, Hercules, CA) and Baird Parker agar (Becton, Dickinson and Company) for the isolation of MRSA and total *S. aureus,* respectively. Plates were incubated at 37°C for 24 hr. Resulting black colonies with halos on Baird Parker agar and hot pink colonies on MRSASelect were considered presumptive *S. aureus* and MRSA, respectively. These colonies were purified on Brain Heart Infusion (BHI) agar (Becton, Dickinson and Company) and archived in Brucella broth (Becton, Dickinson and Company) with 15% glycerol at –80°C. For quality control and quality assurance throughout the isolation process, *S. aureus* ATCC 43300 [American Type Culture Collection (ATCC), Manassas, VA] was used as a positive control and phosphate-buffered saline was used as a negative control.

*Identification. S. aureus* and MRSA were confirmed using Gram stain, the coagulase test (Becton, Dickinson and Company), the catalase test, and polymerase chain reaction (PCR). DNA extraction was carried out using the MoBio UltraClean® Microbial DNA Isolation Kit (Mo Bio Laboratories, Carlsbad, CA) following the manufacturer’s recommendations. For confirmation of *S. aureus*, we carried out PCR amplification of the *S. aureus*-specific *nuc* gene using NUC1 and NUC2 primers (Fang and Hedin 2003). For MRSA differentiation, we performed PCR amplification targeting the *mecA* gene, which encodes for methicillin resistance, using ECA1 and MECA2 primers, as previously described by Fang and Hedin (Brakstad et al. 1992; Fang and Hedin 2003; Smyth et al. 2001). The method was modified by including an internal control, using primers targeting the 16S rDNA genes, in a multiplex PCR assay (Edwards et al. 1989). PCR amplification consisted of an initial denaturing step of 95°C for 3 min, followed by 34 cycles of denaturing at 94°C for 30 sec, annealing at 55°C for 30 sec, and extension at 72°C for 30 sec, with a final extension at 72°C for 5 min.

*Antimicrobial susceptibility testing.* We performed antimicrobial susceptibility testing on all PCR-confirmed MRSA (*n* = 240) and MSSA (*n* = 119) isolates using the Sensititre® microbroth dilution system (Trek Diagnostic Systems Inc., Cleveland, OH) in accordance with the manufacturer’s instructions. Overnight cultures were transferred to sterile demineralized water (Trek Diagnostic Systems) to achieve a 0.5 McFarland standard. Then, 30 µL of each suspension was transferred to sterile cation-adjusted Mueller Hinton broth (Trek Diagnostic Systems) and 50 µL of the broth solution was then dispensed into GPN3F minimal inhibitory concentration (MIC) plates (Trek Diagnostic Systems Inc.) with the following antibiotics: erythromycin (ERY; 0.25–4 µg/mL), clindamycin (CLI; 0.12–2 µg/mL), quinupristin/dalfopristin (SYN; 0.12–4 µg/mL), daptomycin (DAP; 0.25–8 µg/mL), vancomycin (VAN; 1–128 µg/mL), tetracycline (TET; 2–16 µg/mL), ampicillin (AMP; 0.12–16 µg/mL), gentamicin (GEN; 2–16, 500 µg/mL), levofloxacin (LEVO; 0.25–8 µg/mL), linezolid (LZD; 0.5–8 µg/mL), ceftriaxone (AXO; 8–64 µg/mL), streptomycin (STR; 1,000 µg/mL), penicillin (PEN; 0.06–8 µg/mL), rifampin (RIF; 0.5–4 µg/mL), gatifloxacin (GAT; 1–8 µg/mL), ciprofloxacin (CIP; 0.5–2 µg/mL), trimethoprim/sulfamethoxazole (SXT; 1/19–4/76 µg/mL), and oxacillin+2%NaCl (OXA+; 0.25–8 µg/mL). *Enterococcus faecalis* ATCC 29212 and *S. aureus* ATCC 29213 strains were used for quality control. MICs were recorded as the lowest concentration of an antimicrobial that completely inhibited bacterial growth [Clinical and Laboratory Standards Institute (CLSI) 2010]. Resistance break points published by the CLSI were used (CLSI 2010). Multidrug resistance (MDR) was defined as resistance to two or more classes of antibiotics.

*SCC*mec *typing.* We used a multiplex PCR assay developed by Milheiriço et al. (2007) to characterize the MRSA isolates (*n* = 240) by SCC*mec* type (Milheiriço et al. 2007; Oliveira and de Lencastre 2002). SCC*mec* strains COL (type I), BK2464 (type II), ANS46 (type III), MW2 (type IVa), HAR22 (type IVh), and HDE288 (type VI) were used as positive controls for SCC*mec* typing.

*PVL screening.* All MRSA isolates, confirmed by possession of the *nuc* and *mecA* genes by PCR and an identifiable SCC*mec* type (*n* = 236), were screened for PVL by PCR of the *pvl* gene according to Strommenger et al. (2008). *S. aureus* ATCC strain 25923 was used as a positive control.

*PFGE.* We performed PFGE on a subset of 22 MRSA isolates. To ensure a diverse, representative subset, isolates were selected using the following criteria: treatment plant, sampling date, SCC*mec* type, and each sampling location that had a positive sample. PFGE was based on the Centers for Disease Control and Prevention (CDC) Laboratory Protocol for Molecular Typing of *S. aureus* by PFGE (CDC 2011). We used *Sma*I (Promega, Madison, WI) to digest genomic DNA. Digested samples were run in 1% SeaKem® Gold agarose gels (Cambrex Bio Science Rockland Inc., Rockland, ME) in 0.5X TBE (tris-borate- EDTA) using a CHEF Mapper (Bio-Rad) for 18.5–19 hr at 200 V, 14°C, and initial and final switch of 5 and 40 sec. Cluster analysis was performed using BioNumerics software v5.10 (Applied Maths Scientific Software Development, Saint-Martens-Latem, Belgium) using Dice coefficient and the unweighted pair-group method. Optimization settings for dendrograms were 1.0% with a position tolerance of 0.95%. Based on the similarity of the control strains, isolates were considered clones if similarity was ≥ 88%. *Salmonella* serotype Braenderup strain H9812 was used as the standard. PFGE strain types were compared with USA types (100, 200, 300, 400, 500, 600, 700, 800, 1000, and 1100).

*Statistical analyses.* Descriptive statistics include the percentages of wastewater samples positive for MRSA (Table 1) and MSSA (Table 2) by WWTP. Because PFGE was not performed on all isolates, statistical analyses of antibiotic resistance data were limited to MRSA (*n* = 84) and MSSA (*n* = 58) isolates expressing unique phenotypic profiles; this allowed us to reduce bias that could be introduced by including clones. Two-sample tests of proportions were performed between MRSA and MSSA isolates with respect to the percent resistance of each group of isolates to each of the 18 tested antibiotics. Analysis of variance was then used to compare the average numbers of antibiotics against which MRSA and MSSA isolates were resistant. In all cases, *p*-values ≤ 0.05 were defined as statistically significant. All statistical analyses were performed using Stata/IC 10 (StataCorp LP, College Station, TX) and SAS 9.2 (SAS Institute Inc., Cary, NC).

## Results

*Occurrence of MRSA.* We detected MRSA at all WWTPs in this study. The distribution of MRSA-positive samples differed by WWTP, sampling date, and sampling location (Table 1). Across all treatment plants sampled, 50% (22/44) of wastewater samples were positive for MRSA: 60% (12/20) of samples from Mid-Atlantic WWTPs, and 42% (10/24) of samples from Midwest WWTPs. Eighty-three percent (10/12) of influent samples from all WWTPs were MRSA-positive; 100% (5/5) from Mid-Atlantic WWTPs and 71% (5/7) from Midwest WWTPs. MRSA was not detected in any tertiary-treated (chlorinated) effluent samples (Table 1). However, MRSA was detected in one effluent sample from Midwest WWTP1 in October 2010 when chlorination was not taking place. Overall, Midwest WWTP2 had the lowest percentage of MRSA-positive wastewater samples, with MSRA detected only in the influent (Table 1). This plant is the only WWTP in the present study that does not use an activated sludge reactor step; instead, it uses a system of lagoons for biological treatment.

*Occurrence of MSSA.* MSSA was also detected at all WWTPs in this study. The distribution of MSSA-positive samples differed by WWTP, sampling date, and sampling location (Table 2). Across all treatment plants sampled, 55% (24/44) of wastewater samples were positive for MSSA: 60% (12/20) of samples from Mid-Atlantic WWTPs and 50% (12/24) of samples from Midwest WWTPs. Eighty-three percent (10/12) of influent samples from all WWTPs were MSSA-positive; 100% from Mid-Atlantic WWTPs and 71% from Midwest WWTPs. MSSA was not detected in tertiary-treated (chlorinated) effluent samples (Table 2). However, MSSA was detected in two effluent samples from Midwest WWTP1 in September and October 2010 when chlorination was not taking place. Of all four WWTPs, Midwest WWTP2 had the lowest percentage of MSSA-positive wastewater samples, and MSSA was detected only in the influent.

**Figure 2:**
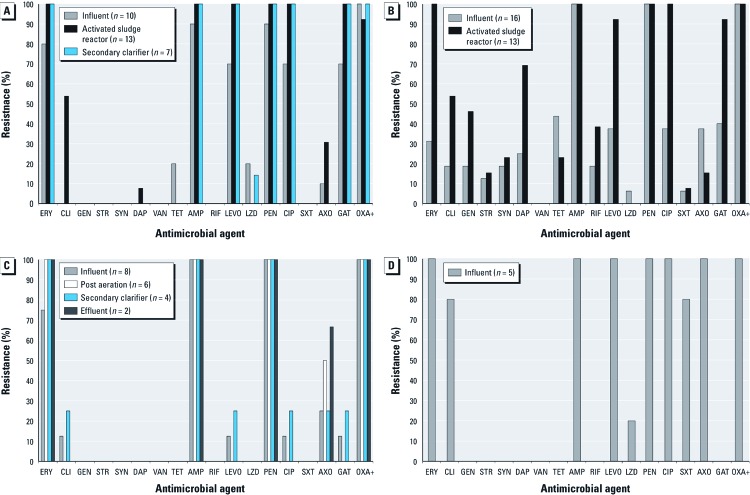
Resistance to antimicrobial agents detected among MRSA isolates at (*A*) Mid-Atlantic WWTP1, (*B*) Mid-Atlantic WWTP2, (*C*) Midwest WWTP1, and (*D*) Midwest WWTP2. The process for each plant is shown in Figure 1.

*Antibiotic resistance patterns.* In total, 240 MRSA isolates were isolated from all of the WWTPs. However, because PFGE was not performed on all isolates, the statistical analyses concerning antibiotic resistance patterns among these isolates were limited to those that could be confirmed as unique (*n* = 84) using phenotypic analyses. The unique MRSA isolates had a median OXA+ MIC of ≥ 16 µg/mL (range, 4 to ≥ 16 µg/mL) and expressed resistance to several antibiotics approved by the U.S. Food and Drug Administration for treating MRSA infections, including TET, CIP, LEVO, GAT, and CLI, as well as LZD and DAP (Figure 2), which are important alternatives to older antibiotics for treating severe MRSA infections (Johnson and Decker 2008).

Antimicrobial resistance patterns among unique MRSA isolates varied by WWTP and sampling location (Figure 2). In general, at both Mid-Atlantic WWTPs and at Midwest WWTP1, the percentage of isolates resistant to individual antibiotics increased or stayed the same as treatment progressed (Figure 2A–2C). At Midwest WWTP2, only influent samples were positive for MRSA, and the majority of these isolates were resistant to most of the tested antibiotics (Figure 2D).

**Figure 3:**
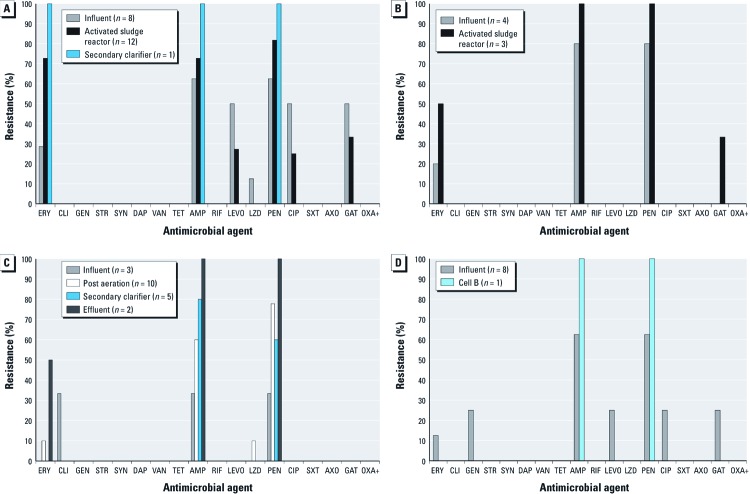
Resistance to antimicrobial agents detected among MSSA isolates at (*A*) Mid-Atlantic WWTP1, (*B*) Mid-Atlantic WWTP2, (*C*) Midwest WWTP1, and (*D*) Midwest WWTP2. The process for each plant is shown in Figure 1.

In total, 119 MSSA isolates were isolated from all WWTPs. Similar to our statistical analyses of MRSA isolates, our analyses of antimicrobial resistance patterns among MSSA isolates were limited to those isolates that could be confirmed as unique (*n* = 58) using phenotypic analyses. Antimicrobial resistance patterns among unique MSSA isolates also varied by WWTP (Figure 3). The percentages of ERY-, AMP- and PEN-resistant unique MSSA isolates at Mid-Atlantic WWTP1 increased as treatment progressed, whereas the percentages of isolates resistant to the fluoroquinolones (LEVO, CIP, and GAT) decreased from influent to activated sludge reactor samples (Figure 3A). At Mid-Atlantic WWTP2, the percentages of ERY-, AMP-, PEN-, and GAT-resistant MSSA isolates increased from influent to activated sludge reactor samples (Figure 3B). Similarly, among Midwest WWTP1 and Midwest WWTP2 MSSA, resistance to AMP and PEN increased as treatment progressed (Figure 3C,D).

In terms of resistance among the groups of isolates, a greater percentage of MRSA isolates than MSSA isolates were resistant to the following 14 antibiotics: ERY, CLI, STR, SYN, DAP, TET, AMP, RIF, LEVO, PEN, CIP, AXO, GAT, and OXA+ (Table 3). MRSA isolates were resistant to more antimicrobials (on average 6.94) than were MSSA isolates (on average 2.26) (*p* < 0.001).

**Table 3:** Percentage of MRSA and MSSA isolates resistant to each tested antibiotic, compared using two-sample tests of proportions.

	Percentage of resistant isolates		*p*-Value (one-sided)
Antibiotic	MRSA	MSSA	
ERY (erythromycin)	82.14 (69/84)	28.57 (16/56)	< 0.0001
CLI (clindamycin)	27.38 (23/84)	1.72 (1/58)	< 0.0001
GEN (gentamicin)	10.84 (9/83)	3.45 (2/58)	0.0537
STR (streptomycin)	4.76 (4/84)	0 (0/58)	0.0459
SYN (quinupristin/dalfopristin)	7.14 (6/84)	0 (0/58)	0.0188
DAP (daptomycin)	16.67 (14/84)	0 (0/58)	0.0005
VAN (vancomycin)	0 (0/83)	0 (0/57)	—
TET (tetracycline)	14.29 (12/84)	0 (0/58)	0.0013
AMP (ampicillin)	98.81 (83/84)	68.97 (40/58)	< 0.0001
RIF (rifampicin)	9.76 (8/82)	0 (0/58)	0.0071
LEVO (levofloxacin)	63.41 (52/82)	15.79 (9/57)	< 0.0001
LZD (linezolid)	5.95 (5/84)	3.45 (2/58)	0.2494
PEN (penicillin)	98.81 (83/84)	73.21 (41/56)	< 0.0001
CIP (ciprofloxacin)	63.10 (53/84)	15.79 (9/57)	< 0.0001
SXT (trimethoprim/sulfamethoxazole)	2.38 (2/84)	0 (0/58)	0.1184
AXO (ceftriaxone)	30.49 (25/82)	0 (0/58)	< 0.0001
GAT (gatifloxacin)	62.65 (52/83)	18.97 (11/58)	< 0.0001
OXA+ (oxacillin+2%NaCl)	98.81 (83/84)	0 (0/58)	< 0.0001

**Figure 4:**
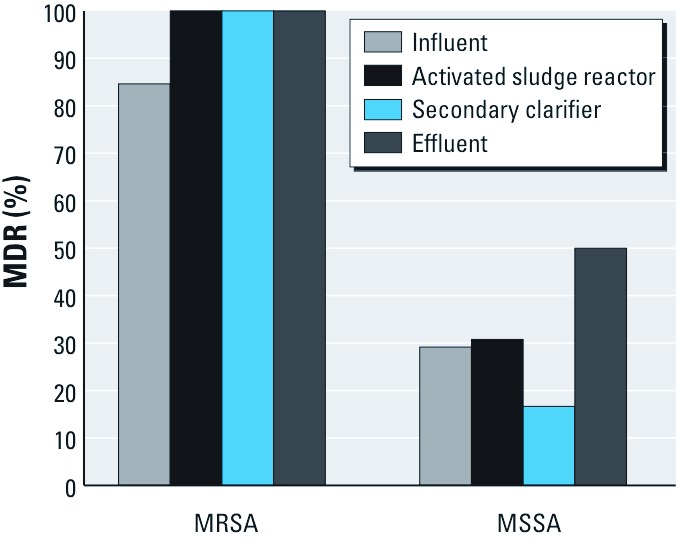
Percentage of multidrug-resistant (resistant to two or more classes of antibiotics) MRSA and MSSA isolates from all WWTPs, by wastewater treatment step.

*Multidrug resistance.* Of phenotypically unique MRSA isolates from all WWTPs, 93% (78/84) were MDR, whereas 29% (17/58) of unique MSSA isolates from all WWTPs were MDR. The summary of MDR MRSA and MSSA by sampling location (across all plants) is shown in Figure 4.

*SCC*mec *typing.* SCC*mec* types II and IV were identified among the MRSA isolates (Table 4). Overall, 83% (199/240) of the MRSA isolates were type IV and 15% (37/240) were type II. For all WWTPs, except Mid-Atlantic WWTP1, only one SCC*mec* type was identified at each plant (Table 4). Four isolates (2%) displayed resistance to OXA+ in antimicrobial susceptibility testing, but did not have the *mecA* band in the Fang and Hedin PCR multiplex or the *mecA* band in the SCCmec PCR multiplex.

**Table 4:** Number (%) of MRSA isolates recovered from wastewater by SCCmec type and by possession of the pvl gene.

Sampling location	SCC*mec* type^a^			PVL-positive^b^
	Type II	Type IV	No *mecA*	
Mid-Atlantic WWTP1 (n = 100)
Influent (n = 40)	0 (0)	40 (100)	0 (0)	28 (70)
Activated sludge reactor (n = 40)	13 (33)	27 (68)	0 (0)	25 (63)
Secondary clarifier (n = 20)	0 (0)	19 (95)	1 (5)	18 (95)
Effluent (n = 0)	0 (0)	0 (0)	0 (0)	0 (0)
Total (n = 100)	13 (13)	86 (86)	1 (1)	71 (72)
Mid-Atlantic WWTP2 (n = 47)
Influent (n = 20)	0 (0)	20 (100)	0 (0)	9 (45)
Activated sludge reactor (n = 27)	0 (0)	27 (100)	0 (0)	26 (96)
Secondary clarifier (n = 0)	0 (0)	0 (0)	0 (0)	0 (0)
Effluent (n = 0)	0 (0)	0 (0)	0 (0)	0 (0)
Total (n = 47)	0 (0)	47 (100)	0 (0)	35 (75)
Midwest WWTP1 (n = 69)				
Influent (n = 22)	0 (0)	19 (86)	3 (14)	9 (47)
Post aeration (n = 21)	0 (0)	21 (100)	0 (0)	20 (95)
Secondary clarifier (n = 13)	0 (0)	13 (100)	0 (0)	13 (100)
Effluent (n = 13)	0 (0)	13 (100)	0 (0)	13 (100)
Total (n = 69)	0 (0)	66 (96)	3 (4)	55 (83)
Midwest WWTP2 (n = 24)
Influent (n = 24 )	24 (100)	0 (0)	0 (0)	0 (0)
Cell B (n = 0)	0 (0)	0 (0)	0 (0)	0 (0)
Effluent (n = 0)	0 (0)	0 (0)	0 (0)	0 (0)
Total (n = 24)	24 (100)	0 (0)	0 (0)	0 (0)
aSCCmec types I, III, V, and VI were not identified in any sample. bPVL PCR was performed only on isolates with the mecA gene.								

*PVL screening.* Among our total MRSA isolates where SCC*mec* type could be confirmed, 68% (161/236) were positive for the *pvl* gene: 72% at Mid-Atlantic WWTP1, 75% at Mid-Atlantic WWTP2, 83% at Midwest WWTP1, and 0% at Midwest WWTP2 (Table 4).

**Figure 5:**
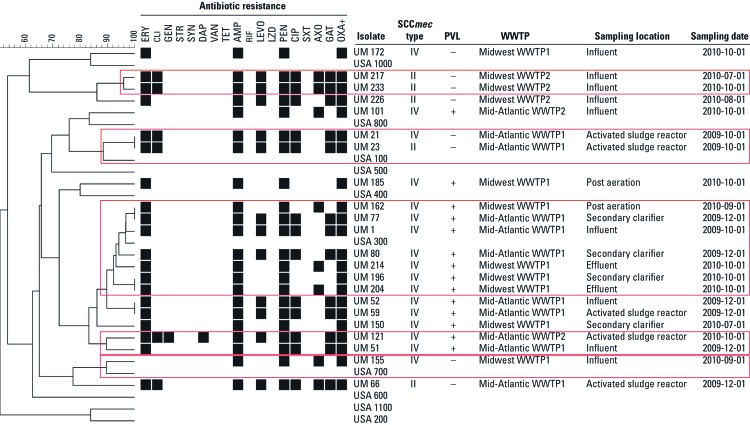
PFGE-based dendrogram, antimicrobial resistance profile, SCC*mec* type, PVL status (positive or negative), and source of a representative subset of MRSA isolates recovered from wastewater. Clusters were based on ≥ 88% similarity and are outlined in red. For antimicrobial resistance phenotypes, black indicates resistance and white indicates intermediate or susceptible. UM, University of Maryland isolate.

*PFGE.* Clusters based on > 88% similarity resulted in 12 unique types among our subset of 22 isolates, suggesting a heterogeneous population among MRSA from U.S. WWTPs (Figure 5). Three different USA types, 100, 300, and 700, were identified. Nine isolates did not match any of the USA types.

## Discussion

*MRSA and MSSA occurrence in U.S. wastewater.* Although MRSA has been identified in WWTPs in Sweden (Börjesson et al. 2009, 2010), to our knowledge, this is the first report of the detection of MRSA at municipal WWTPs in the United States. Fifty percent of total wastewater samples were positive for MRSA, and 55% of total samples were positive for MSSA. Yet, the odds of samples being MRSA-positive decreased as treatment progressed. For example, 10 of 12 (83%) influent samples were MRSA-positive, but only 1 of 12 (8%) effluent samples was MRSA-positive (Table 1). Based on these findings, wastewater treatment seems to reduce the number of MRSA and MSSA isolates released in effluent. However, the few isolates that do survive in effluent might be more likely to be MDR and virulent isolates.

Previous studies conducted in Sweden have also reported a decline in MRSA as wastewater treatment progressed. Specifically, Börjesson et al. (2009) showed that the concentration of MRSA as measured by real-time PCR assays decreased as treatment progressed from approximately 6 × 10^3^ to 5 × 10^2^ *mecA* genes per 100 mL from inlet to outlet, except for a peak in activated sludge reactor samples of 5 × 10^5^ *mecA* genes per 100 mL (Börjesson et al. 2009). On the basis of these findings, we might also expect to see an overall decrease in MRSA concentrations throughout the wastewater treatment process in the United States, except for perhaps a peak in activated sludge. It is also interesting that at Midwest WWTP2, the only WWTP in the study that did not employ an activated sludge step, MRSA was detected only in the influent. The lack of MRSA detected beyond influent at Midwest WWTP2 could be due to the effectiveness of an anaerobic step in the sequencing batch reactor (Figure 1) (Minnigh H, personal communication).

*Cycling of MRSA between humans and the environment.* Our findings also provide evidence that municipal wastewater could serve as a medium for the cycling of CA-MRSA strains between humans and the environment. MRSA has been found at concentrations between 10^4^ and 10^8^ colony-forming units (CFU)/g of fecal material (Wada et al. 2010). PVL-positive strains, SCC*mec* type IV, and USA 300, all of which characterize the majority of the MRSA isolated from wastewater in the present study, have traditionally been associated with CA-MRSA (Gorwitz et al. 2008; Seybold et al. 2006). The high prevalence of PVL-positive CA-MRSA in the U.S. population compared with those in other countries could explain the high percentage of PVL-positive MRSA isolates in wastewater in the present study (Seybold et al. 2006; Tristan et al. 2007). The association of PVL-positive MRSA and CA-MRSA with skin and soft tissue infections could also explain the occurrence of PVL-positive MRSA isolates in wastewater samples in the present study, because MRSA could be shed in showers at concentrations of approximately 1.4 × 10^4^–1.0 × 10^5^ CFU/person (Lina et al. 1999; Plano et al. 2011). The large cluster of MRSA isolates we recovered that were PVL-positive and showed similarity to USA 300 is concerning because both USA 300 strains —which are typically resistant to erythromycin and β-lactam antibiotics—and the *pvl* gene are associated with increased virulence, severe bloodstream infections, and necrotizing pneumonia (Gorwitz et al. 2008; Lina et al. 1999; McDougal et al. 2003).

Moreover, the abundance of SCC*mec* type IV among the recovered MRSA isolates could indicate superior survival characteristics, namely the lower energy cost of SCC*mec* type IV carriage (Börjesson et al. 2010). SCC*mec* type IV strains that we recovered appeared to persist longer in the wastewater treatment process than type II strains. However, this phenomenon warrants further investigation because our results are based on only one WWTP (Mid-Atlantic WWTP1), and a previous study found that SCC*mec* types were not significantly associated with MRSA survival (Levin-Edens et al. 2011).

Four isolates that did not have the *mecA* band in SCC*mec* typing but were found to be OXA+ resistant through antimicrobial susceptibility testing could have the novel *mecA* homolog, MRSA-LGA 251, as identified by García-Álvarez et al. (2011). Interestingly, three of these four isolates were from Midwest WWTP1, which is surrounded by animal production facilities. García-Álvarez et al. (2011) detected the novel *mecA* homolog in bovine MRSA, although the original source of MRSA-LGA 251 is still under investigation (García-Álvarez et al. 2011). Because traditional *mecA* primers do not detect this homolog (García-Álvarez et al. 2011), there could be an even greater number of wastewater samples containing MRSA than were detected in the present study. However, it was beyond the scope of the present study to further assess the wastewater samples for the presence of MRSA-LGA 251.

*Public health implications.* Our findings raise potential public health concerns for WWTP workers and individuals exposed to reclaimed wastewater. WWTP workers could potentially be exposed to MRSA and MSSA through several exposure pathways, including dermal and inhalation exposures. However, few studies have evaluated microbial exposures among WWTP workers. Mulloy (2001) summarized findings of exposures to *Leptospira*, hepatitis A, and bacterial enterotoxins and endotoxins among WWTP workers (Mulloy 2001). Yet, to our knowledge, no studies have evaluated MRSA or MSSA carriage rates among these populations. Encouraging frequent handwashing and the use of gloves among WWTP workers could reduce the potential risks associated with possible MRSA exposures.

Other individuals who are exposed to reclaimed secondary wastewater, including spray irrigators and people living near spray irrigation sites, could be potentially exposed to MRSA and MSSA. No federal regulations exist for wastewater reuse from either secondary or tertiary facilities, although the U.S. Environmental Protection Agency (EPA) has issued water reuse guidelines (U.S. EPA 2004). States determine whether to develop regulations or guidelines to oversee the use of reclaimed wastewater within their boundaries, and most state guidelines allow secondary effluent to be used for certain reuse applications, including spray irrigation of golf courses, public parks, and agricultural areas (U.S. EPA 2004). In the present study, we detected MRSA and MSSA in unchlorinated effluent from Midwest WWTP1, a WWTP with only seasonal chlorination (it could be defined as a secondary treatment plant during periods when chlorine is not applied). Our findings suggest that implementing tertiary treatments for wastewater that is intended for reuse applications could reduce the potential risk of MRSA exposures among individuals who are working on or living by properties sprayed with reclaimed wastewater.

*Limitations.* There are some notable limitations of this study. First, the number and timing of sampling events and samples collected at each WWTP was not the same because of access issues at some of the plants. Second, enrichment of the samples preempted our ability to report concentrations of MRSA and MSSA in wastewater. Finally, because PFGE was performed on a representative subset of all MRSA isolates, the true heterogeneity of the MRSA isolates contained in the wastewater samples may have been underestimated. On the other hand, MRSA strains have evolved from a small number of clonal strains, so the likelihood of isolating MRSA with phenotypic and genetic similarities during our isolation procedure was high (Enright et al. 2002; Fang and Hedin 2003; Oliveira et al. 2002). However, the goal of the present study was to evaluate the occurrence of MRSA at WWTPs in the United States and, even if clones were selected, the findings concerning the presence and types of MRSA at the four WWTPs are still accurate.

## Conclusions

To our knowledge, our study is the first to demonstrate the occurrence of MRSA in U.S. municipal wastewater. Although tertiary wastewater treatment may effectively reduce MRSA in wastewater, secondary-treated wastewater (unchlorinated) could be a potential source of exposure to these bacteria in occupational settings and reuse applications. Because of increasing use of reclaimed wastewater, further study is needed to evaluate the potential risk of antibiotic-resistant bacterial infections from exposure to treated wastewater.
